# A novel TRPM7/*O*-GlcNAc axis mediates tumour cell motility and metastasis by stabilising c-Myc and caveolin-1 in lung carcinoma

**DOI:** 10.1038/s41416-020-0991-7

**Published:** 2020-07-20

**Authors:** Sudjit Luanpitpong, Napachai Rodboon, Parinya Samart, Chanida Vinayanuwattikun, Siwaporn Klamkhlai, Pithi Chanvorachote, Yon Rojanasakul, Surapol Issaragrisil

**Affiliations:** 1 Siriraj Center of Excellence for Stem Cell Research, Faculty of Medicine Siriraj Hospital, Mahidol University, Bangkok, Thailand; 2 Department of Immunology, Faculty of Medicine Siriraj Hospital, Mahidol University, Bangkok, Thailand; 3 Department of Medicine, Division of Medical Oncology, Faculty of Medicine, Chulalongkorn University and The King Chulalongkorn Memorial Hospital, Bangkok, Thailand; 4 Department of Pathology, Faculty of Medicine Siriraj Hospital, Mahidol University, Bangkok, Thailand; 5 Department of Pharmacology and Physiology, Faculty of Pharmaceutical Sciences, Chulalongkorn University, Bangkok, Thailand; 6 WVU Cancer Institute and Department of Pharmaceutical Sciences, West Virginia University, Morgantown, WV USA; 7 Department of Medicine, Division of Hematology, Faculty of Medicine Siriraj Hospital, Mahidol University, Bangkok, Thailand; 8 Bangkok Hematology Center, Wattanosoth Hospital, BDMS Center of Excellence for Cancer, Bangkok, Thailand

**Keywords:** Post-translational modifications, Cancer metabolism, Cell invasion, Oncogenesis

## Abstract

**Background:**

Calcium is an essential signal transduction element that has been associated with aggressive behaviours in several cancers. Cell motility is a prerequisite for metastasis, the major cause of lung cancer death, yet its association with calcium signalling and underlying regulatory axis remains an unexplored area.

**Methods:**

Bioinformatics database analyses were employed to assess correlations between calcium influx channels and clinical outcomes in non-small cell lung cancer (NSCLC). Functional and regulatory roles of influx channels in cell migration and invasion were conducted and experimental lung metastasis was examined using in vivo live imaging.

**Results:**

High expression of TRPM7 channel correlates well with the low survival rate of patients and high metastatic potential. Inhibition of TRPM7 suppresses cell motility in various NSCLC cell lines and patient-derived primary cells and attenuates experimental lung metastases. Mechanistically, TRPM7 acts upstream of *O*-GlcNAcylation, a post-translational modification and a crucial sensor for metabolic changes. We reveal for the first time that caveolin-1 and c-Myc are favourable molecular targets of TRPM7/*O*-GlcNAc that regulates NSCLC motility. *O*-GlcNAcylation of caveolin-1 and c-Myc promotes protein stability by interfering with their ubiquitination and proteasomal degradation.

**Conclusions:**

TRPM7/*O*-GlcNAc axis represents a potential novel target for lung cancer therapy that may overcome metastasis.

## Background

Calcium is a ubiquitous secondary messenger that initiates various signal transduction cascades and influences numerous signalling pathways, controlling fundamental cellular processes, such as cell proliferation, differentiation and cell death.^1–3^ Since these processes are relevant to tumorigenesis, alterations of calcium signalling and homeostasis are thought, if not have been shown, to be implicated in tumorigenesis and tumour progression. Changes in cytosolic free calcium (Ca^2+^) concentrations in response to various extracellular stimuli are typically fuelled by a combination of calcium influx through specialised Ca^2+^-permeable channels and release of Ca^2+^ from intracellular stores—usually endoplasmic reticulum (ER). Intracellular calcium signalling has been observed to be a complex system containing a myriad of proteins, also known as Ca^2+^ toolkit, that are able to bind to Ca^2+^ for their coupling to downstream signalling pathways and finally switching it off by removing the Ca^2+^ from the cytosol.^1^ In the context of cancers, major rearrangement of Ca^2+^ channels by means of changes in their expression and/or function when compared to normal cells has been reported, but there is very limited data concerning these channels and cancer cell motility, particularly in lung cancer.^4–6^

Lung cancer is the leading cause of cancer death that kills more than one million people worldwide each year, due largely to diagnosis at late stages with local or distant organ metastasis.^7^ Cancer cell migration and invasion into surrounding tissues through basement membranes are an initial step in metastasis, allowing the dissemination of primary tumour cells into blood circulation to establish new or secondary tumour sites.^8,9^ Recent studies have emphasised that Ca^2+^ channels that are associated with aggressive cancer behaviours are primarily non-voltage-activated channels and belong to transient receptor potential (TRP) superfamily and Orai family.^10–12^ The TRP proteins that are frequently modified during cancer progression include TRP canonical (TRPC1 and 6), TRP vanilloid (TRPV2 and 6) and TRP melastatin (TRPM7 and 8). Among those, TRPM7 has earlier been shown to be aberrantly expressed in lung adenocarcinoma and squamous cell lung carcinoma and linked to cancer stem cell subpopulation and its carcinogenic potential.^13^ Orai1, through its interaction with ER Ca^2+^ sensor protein STIM1 following depletion of internal Ca^2+^ store, activated store-operated calcium entry at the plasma membrane.^10,14^ Using bioinformatics database, we examined the association between *TRPM7*, *ORAI1* and *STIM1* messenger RNA (mRNA) expression and clinical prognosis in non-small cell lung cancer (NSCLC). We found that high expression of *TRPM7* and *ORAI1* correlate well with low survival rates of NSCLC patients, while patients with high expression of *STIM1* exhibit a similar survival to those with lower expression. Gene repression by CRISPR/Cas9 system revealed an essential role of TRPM7, and to a lesser extent Orai1 and STIM1, in cell motility, leading us to the further investigation of TRPM7 functions, signalling cascades and downstream targets.

Metabolic reprogramming is an emerging hallmark of cancers that occurs during both malignant transformation and tumour development.^15,16^ *O*-GlcNAcylation is an essential post-translational modification (PTM) that uses the final product of nutrient flux UDP-GlcNAc through the hexosamine biosynthetic pathway. As this pathway integrates amino acids, carbohydrates, fatty acids, nucleotides and energy metabolism, the extent of *O*-GlcNAcylation generally reflect the global metabolic dynamics in the cells.^17,18^ Herein, we found that *O*-GlcNAcylation plays a key regulatory role in coordinating calcium signalling via TRPM7 channel, which induces cell migration and invasion in vitro and experimental lung metastasis in vivo. We further unveiled c-Myc and caveolin-1 (Cav-1) as favourable protein-specific *O*-GlcNAcylation that contribute to the motility changes. Our findings suggest a crosstalk between calcium signalling and dysregulated metabolism in cancer cells that could be important in understanding lung cancer progression and metastasis, and may have clinical utility for targeted therapy of lung and other cancers whose aetiology are dependent on abnormal Ca^2+^ influx and *O*-GlcNAcylation.

## Methods

### Survival analysis

Kaplan–Meier survival plots according to the expression of *TRPM7*, *ORAI1* and *STIM1* assessed by gene chip microarrays were generated using the Kaplan–Meier plotter (http://kmplot.com/analysis/) containing clinical data of 2437 lung cancer patients. Only the data from JetSet best probe, which selects the optimal probe set for each gene, was included in the analysis.^19,20^

### Differential gene expression analysis

mRNA expression of *TRPM7* from 109 lung carcinoma tissues were from Bittner’s dataset in Oncomine^TM^ bioinformatics database (https://www.oncomine.org/resource/login.html), which were analysed on Affymetrix U133 Plus 2.0 microarrays and grouped according to their TNM staging into N0, N1 and N2, due to the availability of data. *MGEA5* expression in lung adenocarcinoma and normal tissues were from Su’s dataset, which were analysed on Human Genome U133A Array, as previously described.^21^

### Cell culture and patient-derived primary cancer cells

National Cancer Institute (NCI) lung cancer cell lines, NCI-H292, NCI-H460, A549 and NCI-H23 cells, were obtained from American Type Culture Collection (ATCC, Manassas, VA) and cultured in RPMI-1640 medium containing 10% foetal bovine serum (FBS), 100 U/mL penicillin and 100 μg/mL streptomycin. Cells were maintained in a humidified atmosphere of 5% CO_2_ environment at 37 °C. Patient-derived primary cells were obtained from pleural fluids, which were collected aseptically and heparinised after informed consent and after approval by the Ethics Committee of the Faculty of Medicine, Chulalongkorn University (IRB 365/62).

### Lentiviral production and CRISPR/Cas9-mediated gene knockdown

Lentiviral plasmids carrying guided RNA (gRNA) sequence against human *TRPM7* and SpCas9-blasticidin resistance were kind gifts of Profs. John Doench and David Root (Addgene #76111) and Prof. Feng Zhang (Addgene #52962).^22,23^ All-in-one lentiviral plasmids carrying SpCas9-puromycin resistance and gRNA sequence against human *ORAI1*, *STIM1* and *OGT* were from GenScript (Piscataway, NJ). Lentivirus production was performed using HEK293T packaging cells (ATCC) in conjunction with pCMV.dR8.2 dvpr lentiviral packaging and pCMV-VSV-G envelope plasmids (Addgene #8454 and 8455).^24^ Cells were incubated with lentiviral particles in the presence of hexadimethrine bromide for 48 h and the transfected cells were treated with blasticidin (10 µg/mL) or puromycin (1 µg/mL) for 3 weeks and analysed prior to use by Western blotting.

### Pharmacological inhibition of TRPM7

To study the dose–response pattern of TRPM7 inhibition, pharmacological inhibition of TRPM7 was performed in conjunction with CRISPR/Cas9-mediated genetic manipulation of *TRPM7* using 2-aminoethyl diphenylborinate (2-APB). 2-APB is a general TRP channel blocker that has been shown to inhibit TRPM7 currents in a dose-dependent manner,^25–27^ although this inhibitor is not only specific to TRPM7.

### Short hairpin RNA-mediated gene knockdown

Lentiviral plasmid carrying short hairpin RNA (shRNA) sequence against human *CAV1* was obtained from Santa Cruz Biotechnology, Inc. (Dallas, TX), while retroviral plasmid carrying shRNA sequences against human *MYC* was a kind gift from Prof. Martin Eilers (Addgene #29435).^28^ Retrovirus production was performed using Platinum-A packaging cell lines (Cell Biolabs, San Diego, CA), while lentivirus production and viral particle incubation were performed as described above.

### Cell migration and invasion assays

Cell migration was determined by wound healing assay. A monolayer of cells was cultured in 24-well plate and then a wound space was made with a 1-mm tip width and allowed to migrate for 24–48 h. Micrographs were taken under a phase-contrast microscope (Eclipse Ti-U with NiS-Elements, Nikon, Tokyo, Japan) and wound spaces were measured. Quantitative analysis of cell migration was performed as previously described.^29^ Invasion assay was performed using a 24-well transwell unit with polycarbonate filters (8-μm pore size) coated with Matrigel (BD Bioscience, San Jose, CA). Without Matrigel coating, the control inserts were used for migration assay as an alternative to the wound healing assay. Filling the lower chamber with normal growth medium containing 10% FBS, tested cells were seeded into the upper chamber in serum-free medium and incubated for 48 h. Non-invading/migrating cells were removed from the upper side of membrane using a cotton swab and cells that invaded/migrated to the underside were stained with 10 μg/mL Hoechst 33342 for 30 min. Inserts were visualised and scored under a fluorescence microscope (Eclipse Ti-U). The raw data for wound healing and transwell assays can be found in Supplementary Tables S1 and S2.

### Intracellular Ca^2+^ detection

Intracellular Ca^2+^ was determined using Fura-2 acetoxymethyl ester (Fura-2 AM) as a selective fluorescent probe. Cells were loaded with Fura-2 AM (4 μM) in Hank’s buffered solution (HBSS) solution containing probenecid (2.5 mM), EGTA (3 mM) and 0.1% bovine serum albumin at 37 °C for 90 min. The cells were then centrifuged, resuspended in HBSS and CaCl_2_ (4 μM) were added for 10 min, followed by an analysis of fluorescence intensity using BD FACSCanto flow cytometry (BD Biosciences, San Jose, CA) at 340-nm excitation and 510-nm emission.

### Western blot analysis

After specific treatments, cells were incubated in a commercial lysis buffer (Cell Signaling Technology, Beverly, MA) and a protease inhibitor mixture (Roche Molecular Biochemicals, Indianapolis, IN) at 4 °C for 30 min. Protein content was analysed using BCA protein assay (Pierce Biotechnology, Rockford, IL) and 50 μg of proteins were resolved under denaturing conditions by sodium dodecyl sulfate-polyacrylamide gel electrophoresis (SDS-PAGE) and transferred onto polyvinylidene fluoride membranes. Membranes were blocked with 5% non-fat dry milk, incubated with appropriate primary antibodies at 4 °C overnight and subsequently incubated with peroxidase-conjugated secondary antibodies for 1 h at room temperature. The immune complexes were analysed by enhanced chemiluminescence detection system on a digital imager (ImageQuant LAS, GE Healthcare, Pittsburgh, PA).

### Co-immunoprecipitation, ubiquitination and *O*-GlcNAcylation

Specific protein was immunoprecipitated using protein G-conjugated agarose beads (Santa Cruz Biotechnology). Briefly, cell lysates (200 μg protein) were incubated with anti-c-Myc or anti-Cav-1 antibody at 4 °C overnight, followed by a 3-h incubation with agarose beads at 4 °C. The immune complexes were washed five times with cold lysis buffer and resuspended in 2× Laemmli sample buffer. They were then separated by SDS-PAGE and analysed for ubiquitination or *O*-GlcNAcylation using an anti-ubiquitin or anti-*O*-GlcNAc (RL2) antibody.

### Xenograft mouse model for experimental lung metastasis

Animal care and experimental procedures described in this study were performed in accordance with the Guidelines for Animal Experiments at West Virginia University (WVU) with the approval of the Institutional Animal Care and Use Committee (#1702005551). Male NOD/SCID gamma mice, strain NOD.Cg-Prkdc^scid^ Il2rg^tm1Wjl^/SzJ (NSG; WVU Transgenic Animal Core Facility, Morgantown, WV), aged 6–8 weeks, median weight 26 g, were maintained under pathogen-free conditions within the institutional animal facility and assigned randomly into two experimental groups. Food and tap water were given ad libitum. A total of 1 × 10^6^ cells labelled with UBC-RFP-T2A-Luciferase dual reporter were injected into mice (five mice per group) via tail vein and mice were inspected daily for any signs of distress. Tumour growth of luciferase-labelled cells was monitored at the time of inoculation (week 0; baseline) and on a weekly basis by using in vivo IVIS imaging (PerkinElmer, Waltham, MA) (see Supplementary Materials for full descriptions). At the end of experiments, mice were euthanised with cervical dislocation under carbon dioxide inhalation and the lungs, liver and other organs were dissected and further analysed for tumour histopathology.

### Tumour histopathology

Isolated lung tissues from tumour-bearing mice were formalin fixed and paraffin embedded. The specimens were cut into 5-μm sections and stained with haematoxylin and eosin to define the tumour morphology and cellular structure within the lungs. Tissue sectioning and haematoxylin and eosin staining were performed at the WVU Pathology Laboratory for Translational Medicine, while immunohistochemical (IHC) staining was performed at the Immunopathology Laboratory, Faculty of Medicine Siriraj Hospital.

### Statistical analysis

The data represent means ± s.d. from three or more independent experiments as indicated. Statistical analysis was performed by two-sided Student’s *t* test or one-way analysis of variance, followed by a Bonferroni post-test at a significance level of *p* < 0.05.

## Results

### Higher *TRPM7* expression associates to poorer prognosis of lung carcinoma patients

**Fig. 1:**
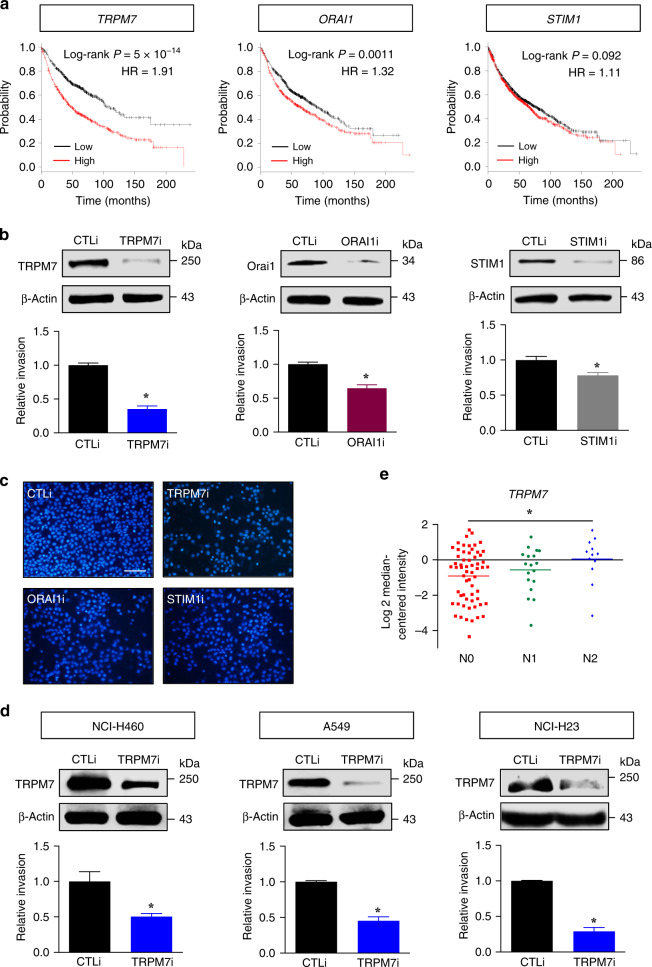
*TRPM7* expression is correlated to poor prognosis and progression of NSCLC. **a** Kaplan–Meier survival plots of NSCLC patients from the gene microarrays generated using the Kaplan–Meier plotter according to the level of *TRPM7*, *ORAI1* and *STIM1*. Overall survival of patients with highest expression (cut-off: median, red line) is compared to those with lowest expression (black line) (*n* = 2437). **b**, **c** NCI-H292 cells were genetically knocked down with TRPM7 (TRPM7i), Orai1 (ORAI1i), STIM1 (STIM1i) or control (CTLi) gRNAs in the CRISPR/Cas9 system. **b** (Upper) TRPM7, Orai1 and STIM1 levels were analysed by Western blotting. (Lower) Cell invasion was evaluated by the transwell assay, where the penetrating cells were stained by Hoechst 33342 dye, quantified and reported as a ratio to CTLi cells. Data are mean ± s.d. (*n* = 4). *p < 0.05 versus CTLi cells; two-sided Student’s *t* test (see also Supplementary Table S1 for raw data). **c** Representative fluorescence micrographs of invading cells stained with Hoechst 33342 dye are shown. Scale bar = 100 μm. **d** Analysis of cell invasion in TRPM7 knockdown (TRPM7i) and control (CTLi) NCI-H460, A549 and NCI-H23 cells by the transwell assay. (Upper) Western blot analysis of TRPM7 level. (Lower) Quantitative analysis of invading cells stained with Hoechst 33342 dye (see also Supplementary Fig. S1 for additional micrographs). Data are mean ± s.d. (*n* = 4). *p < 0.05 versus CTLi cells; two-sided Student’s *t* test. **e** Differential expression of *TRPM7* in normal and lung carcinoma tissues in Bittner’s dataset according to their TNM staging (*n* = 109). *p < 0.05 versus N0; two-sided Student’s *t* test.

Altered calcium signalling often involves an aberrant expression, cellular localisation and function of Ca^2+^ channels, contributing towards specific hallmarks of cancers, such as uncontrolled growth and resisting cell death.^4,6^ We first performed survival analysis in human NSCLC patients using Kaplan–Meier plotter based on mRNA expression of *TRPM7*, *ORAI1* and *STIM1* to assess the clinical significance of these major channels. The population of patients in the database was divided into high and low expression groups using median value to set the cut-off. Figure 1a reveals that the overall survival was significantly reduced in patients with high *TRPM7* and *ORAI1* expression, but not *STIM1*, in comparison with patients with low expression (hazard ratio (HR) = 1.91 and HR = 1.32, respectively), suggesting that TRPM7 and Orai1 may serve as a prognostic factor in lung carcinoma. We hypothesised that TRPM7 and Orai1 may be involved in the regulation of lung cancer metastasis. CRISPR/Cas9 system was used to inhibit TRPM7 and Orai1 in NSCLC NCI-H292 cells and cell invasion, which is a critical step in the metastatic process, was determined by the transwell assay. TRPM7i, and to a lesser extent Orai1 (ORAI1i) and STIM1 (STIM1i), reduced the invasiveness of NCI-H292 cells as compared to CRISPR/Cas9 control (CTLi) (Fig. 1b, c). The inhibitory role of TRPM7 inhibition on cell invasion was similarly observed in NCI-H460, A549 and NCI-H23 cells (Fig. 1d and Supplementary Fig. S1), suggesting the generality of the observed effect. Bioinformatics analysis of an association between *TRPM7* expression and staging of human lung carcinoma revealed that high *TRPM7* correlates well with lymph node metastasis (stage N0 versus N2) (Fig. 1e) and thus further support the role of TRPM7 in the progression of lung cancer.

### Inhibition of TRPM7 and Ca^2+^ influx suppresses cell migration and invasion

**Fig. 2:**
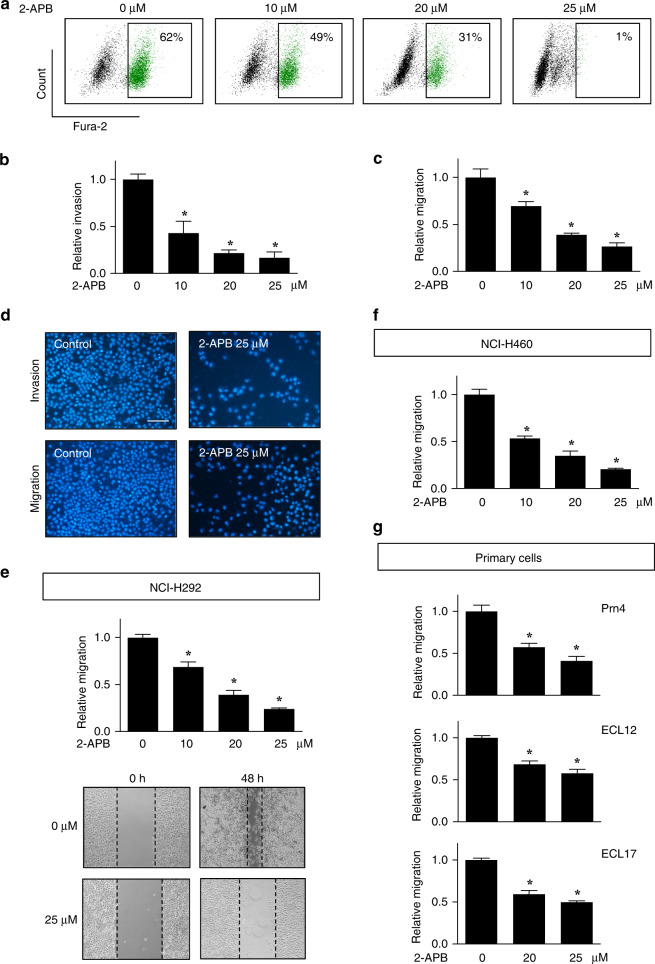
Inhibition of TRPM7 suppresses NSCLC cell migration and invasion. **a** Flow cytometric analysis of intracellular Ca^2+^ in NCI-H292 cells treated with TRPM7 inhibitor 2-APB (0–25 μM) using Fura-2 AM as a specific fluorescent probe (see also Supplementary Fig. S2 for quantitative analysis of intracellular Ca^2+^ by a fluorescence plate reader). **b**–**d** NCI-H292 and NCI-H460 cells were treated with 2-APB (0–25 μM) and cell invasion (**b**) and migration (**c**) were evaluated by transwell assays at 48 h post incubation. Data are mean ± s.d. (*n* = 4). *p < 0.05 versus nontreated cells; two-sided Student’s *t* test. **d** Representative fluorescence micrographs of migrating and invading cells stained with Hoechst 33342 dye are shown. Scale bar = 100 μm. **e**–**g** 2-APB-treated NCI-H292 (**e**), NCI-H460 (**f**) and primary lung cancer (**g**) cells were evaluated for cell migration by wound healing assay. Wound space was visualised at 0 and 24 or 48 h and % change in the space was calculated and reported as a ratio to nontreated cells. Data are mean ± s.d. (*n* = 4). *p < 0.05 versus nontreated cells; two-sided Student’s *t* test (see also Supplementary Table S2 for raw data).

To determine further the causal relationship between TRPM7 function and lung carcinoma cell motility, NSCLC NCI-H292 and NCI-H460 cells were incubated with various concentrations of a non-specific inhibitor of TRPM7, 2-APB, and its effects on Ca^2+^ influx, cell migration and invasion were evaluated. We identified and used the appropriate noncytotoxic concentrations of 2-APB, which is up to 25 μM, to ensure that the observed effects are not due to the cytotoxicity or reduced proliferation. Free intracellular Ca^2+^ in response to 2-APB treatment was evaluated by flow cytometry using Fura-2 AM as a specific fluorescence probe. Figure 2a shows that 2-APB (10–25 μM) was able to decrease the fluorescence intensity of Ca^2+^-bound Fura-2 under the control level, thus validating its inhibitory effect on Ca^2+^ influx (see also Supplementary Fig. S2). Treatment of NCI-H292 cells with 2-APB (0–25 μM) caused a dose-dependent decrease in cell invasion (Fig. 2b and Supplementary Fig. S3), in agreement with the results observed from *TRPM7* gene manipulation (Fig. 1b). Likewise, cell migration, as evaluated by both transwell and wound healing assays, was dose-dependently decreased by the 2-APB treatment (0–25 μM) in NSCLC cells (Fig. 2c–f and Supplementay Figs. S3 and S4).

Lung cancer patient-derived primary cells were obtained from fresh patient-derived pleural effusion (see Supplementary Table S3 for their clinical characterisation) and similarly evaluated for cell migration by wound healing assay following 2-APB treatment (0–25 μM). Consistent with the findings obtained from NSCLC cell lines, we observed a dose-dependent decrease in cell migration upon TRPM7 inhibition in multiple primary lung cancer cells (Fig. 2g and Supplementary Fig. S5). Together, these results demonstrate the role of TRPM7 and Ca^2+^ influx in NSCLC cell motility regulation.

### TRPM7 regulates cell motility mainly through *O*-GlcNAcylation

**Fig. 3:**
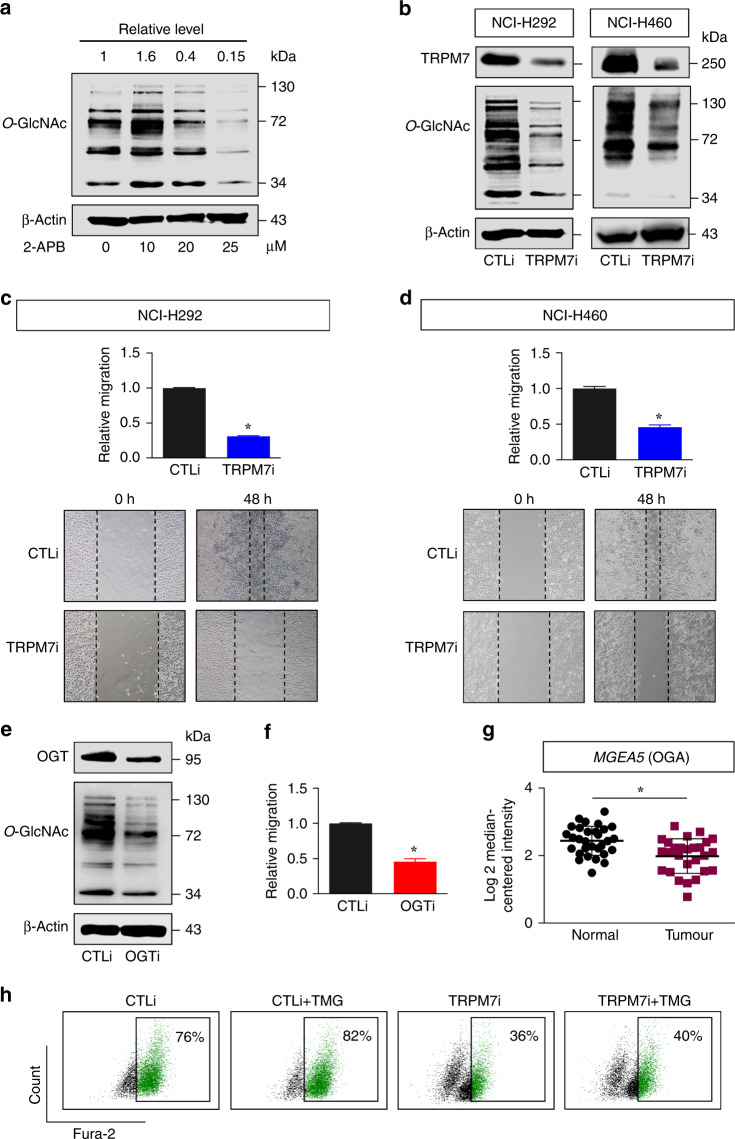
TRPM7 regulates *O*-GlcNAcylation in NSCLC cells. **a** Western blot analysis of global *O*-GlcNAc level in NCI-H292 cells treated with 2-APB (0–25 μM). β-Actin was used as a loading control. **b** Western blot analysis of TRPM7 and *O*-GlcNAc levels in TRPM7 knockdown (TRPM7i) NCI-H292 and NCI-H460 cells. **c**, **d** Cell migration of TRPM7i NCI-H292 (**c**) and NCI-H460 (**d**) cells was compared to that of CTLi cells using wound healing assay at 48 h. Plots are relative migration calculated from % change in wound space and normalised to CTLi cells. Data are mean ± s.d. (*n* = 4). *p < 0.05 versus CTLi cells; two-sided Student’s *t* test. **e**, **f** NCI-H292 cells were genetically knocked down with OGT (OGTi) or control (CTLi) gRNAs in CRISPR/Cas9 system (**e**) and cell migration was evaluated by wound healing assay at 48 h (**f**). Plots are relative migration calculated from % change in wound space and normalised to CTLi cells. Data are mean ± s.d. (*n* = 4). *p < 0.05 versus CTLi cells; two-sided Student’s *t* test. Representative micrographs of wound space are shown in Supplementary Fig. S6. **g** Differential expression of *MGEA5* (encoding OGA) in normal and lung carcinoma tissues in the Su’s dataset. *p < 0.05 versus CTLi cells; two-sided Student’s *t* test. See also Supplementary Fig. S7 for Kaplan–Meier survival curve of patients with NSCLC according to *MGEA5* expression. **h** Flow cytometric analysis of intracellular Ca^2+^ in TRPM7i and CTLi cells in the presence or absence of the OGA inhibitor thiamet G (TMG; 10 μM) using Fura-2 AM as a specific fluorescent probe (see also Supplementary Fig. S8 for quantitative analysis of intracellular Ca^2+^ by a fluorescence plate reader).

Hyper-*O*-GlcNAcylation is a general feature of various human malignancies that may contribute to transformed phenotypes. Level of *O*-GlcNAcylation has been suggested to be a regulator of cancer metastasis in human prostate, breast and ovarian cancers.^30–32^ We hypothesised a plausible crosstalk between the calcium signalling and *O*-GlcNAcylation and herein tested this possibility in NSCLC. We observed a remarkable decrease in global *O*-GlcNAcylation in NSCLC cells upon treatment with the TRPM7 inhibitor 2-APB (>10 μM) (Fig. 3a) and upon TRPM7 knockdown (Fig. 3b), suggesting the functional linkage between TRPM7-mediated cell motility and *O*-GlcNAcylation.

*O*-GlcNAcylation is dynamically modulated by two cycling enzymes *O*-GlcNAc transferase (OGT) and *O*-GlcNAcase (OGA) that catalyses and removes *O*-GlcNAc. We next manipulated OGT in NCI-H292 cells by CRISPR/Cas9-mediated gene knockdown to decrease cellular *O*-GlcNAcylation to ascertain its role in cell motility of NSCLC cells. Similar to that observed in TRPM7i cells (Fig. 3c, d), an inhibition of OGT effectively decreased cell migration as compared to control (Fig. 3e, f and Supplementary Fig. S6), thus confirming the promoting role of hyper-*O*-GlcNAcylation in NSCLC motility. The significance of hyper-*O*-GlcNAcylation was further strengthened by an observed low *MGEA5* (encoding OGA) expression in clinical lung tumour specimens as compared to normal lung tissues (Fig. 3g) that were also found to be associated with lower survival rates of patients (Supplementary Fig. S7).

**Fig. 4:**
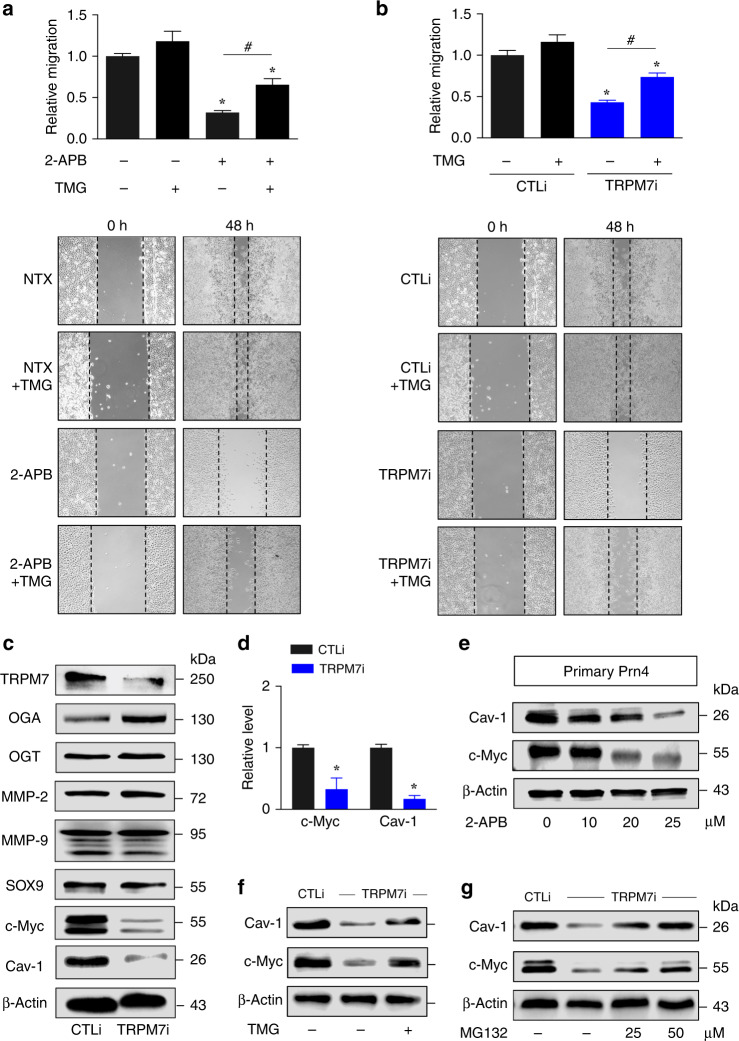
NSCLC cell motility is mediated by TRPM7/*O*-GlcNAc regulatory axis. **a**, **b** Cell migration assay of NCI-H292 cells upon TRPM7 inhibition by 2-APB (20 μM) (**a**) or CRISPR/Cas9 (TRPM7i) (**b**) in the presence or absence of thiamet G (TMG; 10 μM) at 48 h. Plots are relative migration normalised to control cells. Data are mean ± s.d. (*n* = 4). *p < 0.05 versus nontreated or CTLi cells; one-way ANOVA with Bonferroni post-test. ^#^*p* < 0.05 versus 2-APB-treated or TRMP7i cells without TMG; one-way ANOVA with Bonferroni post-test. **c** Western blot analysis of TRPM7, OGA, OGT or key regulators of lung tumour metastasis, including MMP-2, MMP-9, SOX9, c-Myc and Cav-1 in TRPM7i and CTLi cells. **d** Quantitative analysis of c-Myc and Cav-1 by densitometry is shown (see also Supplementary Fig. S9 for quantitative analysis of other proteins and quantitative real-time PCR of *TRPM7*, *MGEA5*, *OGT*, *MYC* and *CAV1* mRNA expression). Plots are fold difference relative to CTLi cells after normalisation to the loading control. Data are mean ± s.d. (*n* = 3). *p < 0.05 versus CTLi cells; two-sided Student’s *t* test. **e** Inhibition of TRPM7 decreases Cav-1 and c-Myc in lung cancer patient-derived primary cells. Primary Prn4 cells were treated with 2-APB (0–25 μM) for 24 h and Cav-1 and c-Myc levels were evaluated by Western blotting. β-Actin was used as a loading control. **f**, **g** Western blot analysis of Cav-1 and c-Myc in TRPM7 knockdown (TRPM7i) and control (CTLi) NCI-H292 cells in the presence or absence of the OGA inhibitor thiamet G (TMG; 10 μM) (**f**) or proteasomal inhibitor MG132 (25–50 μM) (**g**) (see also Supplementay Fig. S11 for quantitative analysis of proteins by densitometry).

To further validate that TRPM7 regulates cell motility through *O*-GlcNAcylation, rescue experiments were conducted in which *O*-GlcNAcylation was induced in TRPM7 knockdown cells and its effects on cell motility and Ca^2+^ influx were examined. Thiamet G, a small-molecule inhibitor of OGA, was used to induce cellular *O*-GlcNAcylation in this study. Intracellular Ca^2+^ level was found to be reduced in TRPM7i cells, but not further changed in thiamet G-treated cells in both TRPM7i and CTLi cells (Fig. 3h; see also Supplementary Fig. S8). Figure 4a shows that although thiamet G had minimal effect on the nontreated control cells, which is likely due to their limited migratory capacity, co-treatment of thiamet G (10 μM) and 2-APB (20 μM) significantly promoted cell migration as compared to 2-APB treatment alone. A similar finding on the rescue of TRPM7i cell migration by thiamet G (10 μM) was also observed (Fig. 4b). Altogether, these data indicate that *O*-GlcNAcylation is a downstream regulator of TRPM7 and Ca^2+^ influx and is involved in cell motility regulation in NSCLC cells.

### Cav-1 and c-Myc are favourable downstream targets of TRPM7/*O*-GlcNAc

To elucidate the underlying mechanisms by which TRPM7 and *O*-GlcNAcylation mediate cell motility, we monitored the levels of various proteins known to be important in metastasis of lung tumour, including matrix metalloproteinases MMP-2 and MMP-9,^33,34^ SOX9,^35^ c-Myc and Cav-1,^36,37^ following TRPM7 inhibition. The results show that MMP-2, MMP-9 and SOX9 were relatively unchanged when comparing TRPM7i and control cells, suggesting their unlikely involvement in the TRPM7/*O*-GlcNAc regulatory axis (Fig. 4c). On the other hand, a striking downregulation of c-Myc and Cav-1 was observed in TRPM7i cells (Fig. 4c, d and Supplementary Fig. S9) as well as in 2-APB-treated primary cells (Fig. 4e), suggesting c-Myc and Cav-1 as potential molecular targets of this regulatory process. It is worth noting here that the precise mechanism of how TRPM7 regulates *O*-GlcNAcylation remains unclear and is likely to be complex and diverse since *O*-GlcNAcylation senses and responds to a wide variety of stimuli and stresses. However, our results indicate that this might involve a modulation of *O*-GlcNAc cycling enzymes, as an increase of OGA and an upregulation of *OGT* were observed in the TRPM7i cells (Supplementary Fig. S9).

### TRPM7 and *O*-GlcNAcylation inhibit Cav-1 and c-Myc degradation

To gain a better insight into how TRPM7 modulates Cav-1 and c-Myc, quantitative real-time PCR was performed to analyse their mRNA expression in TRPM7i and control cells. Supplementary Fig. S9 shows that TRPM7 inhibition had no significant effect on *CAV1* transcription, suggesting that Cav-1 is regulated by TRPM7 at post-translational level. By contrast, a subtle yet significant downregulation of *MYC* was observed in TRPM7i cells, despite a large decrease in c-Myc protein. It is hence conceivable that TRPM7 regulates c-Myc at both transcriptional and post-translational levels.

**Fig. 5:**
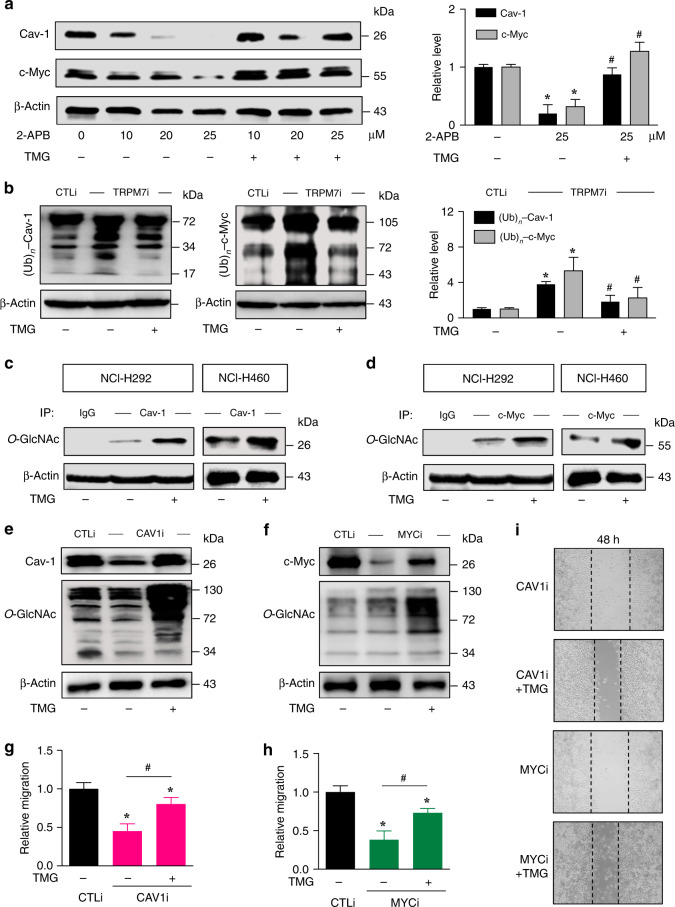
Cav-1 and c-Myc are favourable targets of TRPM7/*O*-GlcNAc regulatory axis. **a** Dose profile of Cav-1 and c-Myc levels in response to 2-APB treatment (0–25 μM) in the presence or absence of thiamet G (TMG; 10 μM), as evaluated by Western blotting. Plots are fold difference relative to nontreated control cells after normalisation to the loading control. Data are mean ± s.d. (*n* = 3). *p < 0.05 versus nontreated cells; one-way ANOVA with Bonferroni post-test. ^#^*p* < 0.05 versus 2-APB-treated cells without TMG; one-way ANOVA with Bonferroni post-test. **b** Analysis of Cav-1 and c-Myc ubiquitination in TRPM7i and CTLi cells in the presence or absence of TMG (10 μM) at 3 h. All cells were treated with MG132 (50 μM) to prevent proteasomal degradation of Cav-1 and c-Myc and cell lysates were prepared and immunoprecipitated using anti-Cav-1 (left) and anti-c-Myc (right) antibodies. β-Actin of IP input lysate was used as a loading control. Data are mean ± s.d. (*n* = 3). *p < 0.05 versus CTLi cells; one-way ANOVA with Bonferroni post-test. ^#^*p* < 0.05 versus TRPM7i cells without TMG; one-way ANOVA with Bonferroni post-test. The efficiency of Cav-1 and c-Myc immunoprecipitation was shown in Supplementary Fig. S12. **c**, **d** Analysis of Cav-1 and c-Myc *O*-GlcNAcylation in NCI-H292 or NCI-H460 cells to confirm that Cav-1 and c-Myc are targets of *O*-GlcNAcylation. Cells were treated with TMG for 6 h and cell lysates were prepared and immunoprecipitated using anti-Cav-1 (**c**) and anti-c-Myc (**d**) antibodies. β-Actin of IP input lysate was used as a loading control. **e**–**i** NCI-H292 cells were genetically knocked down with CAV1 (CAV1i), MYC (MYCi) or control (CTLi) shRNAs. **e**, **f** Western blot analysis of Cav-1, c-Myc and *O*-GlcNAc levels in CAV1i and MYCi cells in the presence or absence of TMG (10 μM) for 24 h. **g**–**i** Cell migration assay of CAV1i, MYCi and CTLi cells in the presence or absence of TMG (10 μM) as evaluated by wound healing assay at 48 h. Plots are relative migration calculated from % change in wound space and normalised to CTLi cells. Data are mean ± s.d. (*n* = 4). *p < 0.05 versus CTLi cells; one-way ANOVA with Bonferroni post-test. ^#^*p* < 0.05 versus CAV1i or MYCi cells without TMG; one-way ANOVA with Bonferroni post-test. Representative micrographs of wound space are shown here (**i**) and in Supplementary Fig. S13. See also Supplementary Fig. S14 for phenotype rescue experiments upon TRPM7 inhibition by Cav-1 and c-Myc overexpression.

*O*-GlcNAcylation is known to influence protein stability and function.^21,38,39^ To directly determine whether *O*-GlcNAcylation of c-Myc and Cav-1 could protect them from TRPM7 inhibition, TRPM7i cells were treated with thiamet G (10 μM) that causes hyper-*O*-GlcNAcylation (Supplementary Fig. S10) and its effect on Cav-1 and c-Myc was evaluated by Western blotting. Figure 4f and Supplementary Fig. S11a shows that thiamet G reversed the repression of Cav-1 and c-Myc by TRPM7 inhibition in TRPM7i cells, thereby confirming the TRPM7/*O*-GlcNAc regulatory axis of Cav-1 and c-Myc. Proteasomal degradation is a major post-translational cellular process that controls protein turnover. To determine whether Cav-1 and c-Myc under TRPM7 regulation are controlled by proteasomal degradation, TRPM7i cells were treated with MG132 for proteasome inhibition. The increase in Cav-1 and c-Myc expression by MG132 treatment suggested proteasomal degradation as an important mechanism of Cav-1 and c-Myc regulation by TRPM7 and *O*-GlcNAcylation (Fig. 4g and Supplementary Fig. S11b). To strengthen this finding, the dose profile of Cav-1 and c-Myc repression in response to 2-APB treatment (0–25 μM), which was substantially rescued by the presence of thiamet G (10 μM), was generated and shown in Fig. 5a.

Given that numerous proteins, including Cav-1 and c-Myc, are marked for proteasomal degradation by ubiquitination,^21,29^ we analysed Cav-1 and c-Myc ubiquitination in TRPM7i and CTLi cells in the presence or absence of thiamet G (10 μM). Figure 5b shows that TRPM7 inhibition induced Cav-1 and c-Myc ubiquitination and that an addition of thiamet G could inhibit their ubiquitination (see also Supplementary Fig. S12). As both *O*-GlcNAcylation and ubiquitination occurs on serine and/or threonine residues of protein, it is plausible that *O*-GlcNAcylation might interfere with Cav-1 and c-Myc ubiquitination and subsequently protect them from proteasomal degradation. Co-immunoprecipitation of Cav-1 and c-Myc with *O*-GlcNAc in NCI-H292 and NCI-H460 cells confirms that Cav-1 and c-Myc are true targets of *O*-GlcNAcylation in NSCLC (Fig. 5c, d). Phenotypically, hyper-*O*-GlcNAcylation by thiamet G was demonstrated to reactivate the migration of NCI-H292 cells following Cav-1 and c-Myc inhibition by shRNA-mediated gene knockdown in agreement with the results of Cav-1, c-Myc and *O*-GlcNAc levels (Fig. 5e–i and Supplementary Fig. S13). Rescue experiments with Cav-1 and c-Myc overexpression were also conducted in TRPM7i cells (Supplementary Fig. S14) to once again validate that Cav-1 and c-Myc are regulated by TRPM7/*O*-GlcNAc axis.

### Inhibition of TRPM7 attenuates lung metastasis

**Fig. 6:**
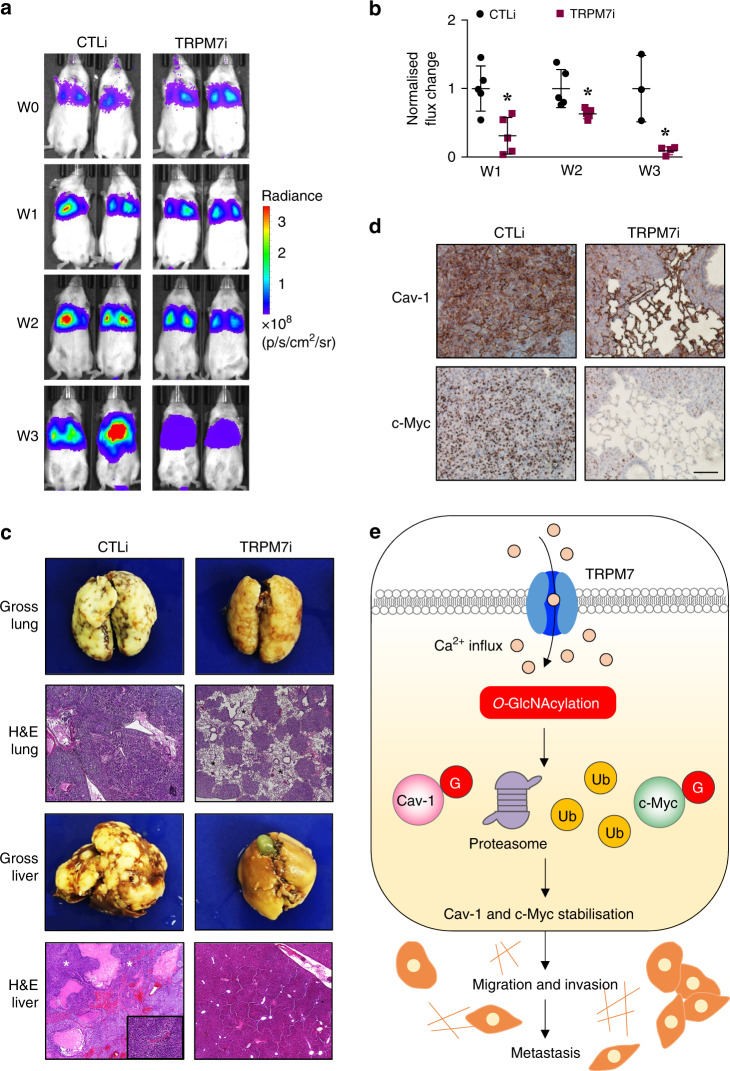
Inhibition of TRPM7 suppresses experimental lung cancer metastasis *in vivo*. Luciferase-labelled TRPM7i or CTLi NCI-H292 cells were injected into NSG mice via tail vein. **a** Representative bioluminescence of mice taken at the time of inoculation (week 0) and at 1, 2 and 3 weeks post injection. **b** Quantitative analysis of bioluminescence signals over time. Plots are fold difference relative to CTLi cells after normalisation to their initial signal at the time of inoculation. Data are mean ± s.d. (*n* = 5). *p < 0.05 versus CLTi cells; two-sided Student’s *t* test. **c** Representative gross pathology and H&E micrographs of lung and liver tissues. (Upper) Asterisks show larger alveolar space without metastatic foci. (Lower) Asterisks show hypercellular neoplastic lesions. **d** IHC analysis of Cav-1 and c-Myc in isolated lungs from mice bearing CTLi and TRPM7i cells. Scale bar = 100 μm (see also Supplementary Fig. S15 for the IHC analysis of TRPM7). **e** A schematic working model for the function of TRPM7/*O*-GlcNAc regulatory axis in NSCLC cell motility and metastasis. TRPM7 regulates Ca^2+^ influx in NSCLC cells, resulting in an increase of *O*-GlcNAcylation of Cav-1 and c-Myc and their stabilisation. G *O*-GlcNAcylation, Ub ubiquitination.

Having demonstrated that TRPM7 acts upstream of *O*-GlcNAcylation, Cav-1 and c-Myc, regulating cell migration and invasion in NSCLC cells, we experimentally verified the involvement of TRPM7 in metastasis using a xenograft mouse model. TRPM7i and control cells were genetically labelled with luciferase and injected into NSG mice via tail vein at the dose of 1 × 10^6^ cells per mouse. Tumour growth was monitored weekly by measuring the luciferase activity associated with the growing tumour cells using IVIS bioluminescence imaging (Fig. 6a). Quantitative analysis of luminescence signals revealed that tumour growth was significantly lower in mice bearing TRPM7i cells relative to control mice at week 1, 2 and 3 post injection (Fig. 6a, b), consistent with the observations of reduced number of experimental lung metastases (Fig. 6c, upper). IHC analysis further revealed the overall diminished intensities of Cav-1 and c-Myc in the isolated TRPM7i lungs (Fig. 6d), due to both lower expression and lower number of infiltrated cells, consistent with the in vitro findings (see also Supplementary Fig. S15). It should be noted that mice were sacrificed after 3 weeks post injection because more than half of the control mice died. Anatomical and histological analyses of the dissected organs in TRPM7i mice showed much less metastatic cells in major organs, such as the liver (Fig. 6c, lower) and kidney (data not shown), as compared to control mice. These data support the in vitro findings and substantiate the critical regulatory role of TRPM7 in tumour metastasis of NSCLC and highlight the potential application of TRPM7 inhibition for metastasis of lung cancer.

## Discussion

Dysregulation of calcium signalling is often deleterious and has been linked to certain hallmarks of cancers. However, how aberrant Ca^2+^ influx contributes to tumorigenesis and tumour progression, in particular metastasis in lung cancer, remains largely an unexplored area. In this study, we found that the Ca^2+^-permeable cation channel TRPM7, which is associated with progression and poor prognosis in lung cancer patients, is a key regulator of NSCLC cell migration and invasion and overall metastatic activity. A novel crosstalk between TRPM7 and *O*-GlcNAcylation was uncovered and their molecular targets for controlling NSCLC motility were subsequently identified as Cav-1 and c-Myc.

Virtually all cellular processes that are crucial for determining cell survival, proliferation and normal tissue homeostasis rely on ion channels. Given that aberrant expression and/or function of ion channels can impair these processes driving normal cells into malignant derivatives, the channels that support malignant behaviours of tumour cells or promote core features of cancers, which include: self-sufficiency in growth signals, insensitivity to anti-growth signals, resistance to apoptosis, limitless replication, sustained angiogenesis, and tissue invasion and metastasis; these are called oncochannels.^40,41^ As calcium is a universal signalling ion regulating a plethora of cellular processes, it is not surprising that numerous Ca^2+^ channels are being recognised as oncochannels.

The role of Ca^2+^ channels in facilitating malignant transformation is better known in cell proliferation. Ca^2+^ homeostasis has been shown to be implicated in the regulation of multiple cell cycle checkpoints, for example, cyclins A and E, with the calcium/calcineurin/NFAT pathway being one of the most recognised pathways.^10,42^ Relatively little is known about the role of Ca^2+^ channels in cell motility, which is a critical step in tumour metastasis. Previous studies have suggested the unique function of TRPM7 in controlling cell migration and invasion of ovarian, breast and prostate cancers, yet the precise underlying mechanisms are largely unknown and may vary depending on cellular context. For example, TRPM7 was reported to promote cell migration and tumour growth of prostate cancer cells through cholesterol-dependent Ca^2+^ entry,^43^ while it induces cell migration of oestrogen receptor-negative metastatic breast cancer cells through its kinase domain independent of Ca^2+^ influx.^44^ In ovarian cancer cells, TRPM7 was shown to influence the assembly of focal adhesions and Akt, Src and p38 signalling associated with cell motility.^45^ We further provide evidence in the present study that the role of TRPM7 in NSCLC cell migration and invasion is likely Ca^2+^ dependent, as the TRPM7i either by 2-APB treatment or CRISPR/Cas9-mediated gene knockdown reduced Ca^2+^ influx (Figs. 2a and 3h) in concomitant with the decrease in cell motility (Figs. 1–3). Notably, the dominant role of Ca^2+^ in NSCLC cell motility was observed upon addition of extracellular Ca^2+^ following ion chelation by EDTA (Supplementary Fig. S16).

Alterations of *O*-GlcNAc cycling enzymes OGT and OGA, as well as an elevated level of *O*-GlcNAcylation, have been observed in various solid cancers, including breast, colon, pancreas, liver and lung.^18,30–32^ We previously demonstrated that hyper-*O*-GlcNAcylation renders NSCLC cells to acquired cisplatin resistance via the regulation of p53 and c-Myc.^21^ We herein extend the knowledge that *O*-GlcNAcylation also regulates NSCLC cell motility, as shown by a decrease in cell motility upon OGT knockdown, which in turn reduces *O*-GlcNAcylation (Fig. 3e, f). TRPM7 was further found to affect *O*-GlcNAcylation in part through the modulation of *O*-GlcNAc cycling enzymes (Figs. 3b and 4 and Supplementary Fig. S9). Most importantly, hyper-*O*-GlcNAcylation by thiamet G was able to rescue the reduced migratory phenotype upon TRPM7 inhibition (Fig. 4a, b), thereby uncovering the novel regulation of NSCLC cell motility through TRPM7/*O*-GlcNAc regulatory axis. In non-cancer-related events, the interplay between calcium signalling and *O*-GlcNAcylation has earlier been reported in glucagon-stimulated liver autophagy and metabolic adaptation.^46^ That is, glucagon induces Ca^2+^ release via an intracellular Ca^2+^ channel InsP3R1 and activates CaMKII, of which phosphorylates OGT and promotes *O*-GlcNAcylation and activation of Ulk1, a key regulator in autophagy initiation.

Cav-1 is an essential structural protein component of the plasma membrane microdomains called caveolae, which is shown to be upregulated in lung carcinoma, associating with their invasiveness and poor survival of patients.^29,36,47^ We previously reported that Cav-1 plays an essential role in cancer stem cell regulation and their aggressive phenotypes, including enhanced cell migration and invasion, in an experimental model of lung tumorigenesis.^48^ Here we demonstrated a concomitant decrease in cell motility and Cav-1 expression upon TRPM7 inhibition, which could be reversed by the reactivation of *O*-GlcNAcylation (Figs. 4, 5), defining the TRPM7/*O*-GlcNAc/Cav-1 pathway. Recently, the crosstalk between *O*-GlcNAcylation and ubiquitination has been identified to cause either increased protein stability or turnover. Hypo-*O*-GlcNAcylation of Cav-1 following TRPM7 inhibition post-translationally was shown to promote its ubiquitination and subsequent proteasomal degradation (Fig. 5). To our knowledge, this is the first demonstration of the regulation of Cav-1 by *O*-GlcNAcylation and ubiquitination.

In lung cancer, a frequent gene amplification or copy number gain of *MYC* was detected in small cell lung cancer in up to 40% of all cases and in NSCLC in human tumour tissues and various animal models.^49^ *MYC* gain is associated with lymph node metastasis and is believed to be a metastasis gene for NSCLC.^37^ Like Cav-1, c-Myc was found in this study to be a favourable target of TRPM7 and *O*-GlcNAcylation in controlling NSCLC cell motility. A crosstalk between *O*-GlcNAcylation and ubiquitination of c-Myc was also observed (Figs. 4, 5). When *O*-GlcNAcylation is repressed by TRPM7 inhibition, c-Myc ubiquitination escalates, leading to increased proteasomal degradation. Previous tumorigenesis and metastasis-promoting gene signalling network based on whole-transcriptome gene expression profiles comparing normal lung epithelial cells versus transformed lung cells revealed *MYC* overexpression and its first-order linkage to *CAV1* at the focal position of the network, indicating the potential complex interaction between c-Myc and Cav-1 in the progression of lung carcinoma.^48^ Having demonstrated that TRPM7 acts upstream of the regulatory axis mediating cell motility via c-Myc and Cav-1, we further showed that TRPM7 inhibition could indeed suppress experimental lung metastases (Fig. 6), advising the potential therapeutic application of TRPM7 inhibition for advanced lung cancer.

In summary, the evidence presented here demonstrates that NSCLC cell motility could be modulated by TRPM7 and Ca^2+^ influx through *O*-GlcNAcylation. Hyper-*O*-GlcNAcylation of Cav-1 and c-Myc stabilises the proteins by interfering with their ubiquitination process and subsequent proteasomal degradation, as schematically summarised in Fig. 6e. Our novel findings on TRPM7/*O*-GlcNAc regulatory axis provide an integrated understanding of how calcium signalling links to *O*-GlcNAc signalling and likely dysregulated metabolism, which could aid in the complex understanding of lung cancer progression and metastasis and lay groundwork for future novel therapeutics. Targeting this regulatory axis and downstream targets could be advantageous for the treatment of advanced lung cancer and other related malignancies.

## Supplementary information

Supplementary information

## Data Availability

The datasets used and/or analysed during the current study are available from the corresponding author on reasonable request. Supplementary information is available at the *British Journal of Cancer* website.
