# Supplementary material for: A novel TRPM7/O-GlcNAc axis mediates tumour cell motility and metastasis by stabilising c-Myc and caveolin-1 in lung carcinoma
Source: Br J Cancer. 2020 Jul 20;123(8):1289–301. doi: 10.1038/s41416-020-0991-7 (PMC7555538; doi:10.1038/s41416-020-0991-7)
Supplement: Supplementary file 1 — Supplementary information [file 41416_2020_991_MOESM1_ESM.pdf]

A novel TRPM7/*O*-GlcNAc axis mediates tumor cell motility and metastasis by stabilizing c-Myc and caveolin-1 in lung carcinoma

Sudjit Luanpitpong[1], Napachai Rodboon[1,9], Parinya Samart[2,9], Chanida Vinayanuwattikun[3], Siwaporn Klamkhlai[4], Pithi Chanvorachote[5], Yon Rojanasakul[6], Surapol Issaragrisil[1,7,8]

[1]Siriraj Center of Excellence for Stem Cell Research, Faculty of Medicine Siriraj Hospital, Mahidol University, Bangkok, Thailand; [2]Department of Immunology, Faculty of Medicine Siriraj Hospital, Mahidol University, Bangkok, Thailand; [3]Division of Medical Oncology, Department of Medicine, Faculty of Medicine, Chulalongkorn University and The King Chulalongkorn Memorial Hospital, Bangkok, Thailand; [4]Department of Pathology, Faculty of Medicine Siriraj Hospital, Mahidol University, Bangkok, Thailand; [5]Department of Pharmacology and Physiology, Faculty of Pharmaceutical Sciences, Chulalongkorn University, Bangkok, Thailand; [6]WVU Cancer Institute and Department of Pharmaceutical Sciences, West Virginia University, Morgantown, WV, USA; [7]Division of Hematology, Department of Medicine, Faculty of Medicine Siriraj Hospital, Mahidol University, Bangkok, Thailand; [8]Bangkok Hematology Center, Wattanosoth Hospital, BDMS Center of Excellence for Cancer, Bangkok, Thailand.

[9]These authors contributed equally to this work.

Running title: TRPM7/*O*-GlcNAc regulatory axis of cell motility

Correspondence: Sudjit Luanpitpong, Siriraj Center of Excellence for Stem Cell Research, Faculty of Medicine Siriraj Hospital, Mahidol University, 2 Siriraj Hospital, Bangkoknoi, Bangkok 10700, Thailand; Tel.: +66 2 419 2907; Email: suidjit@gmail.com.

Supplementary information: Supplementary information accompanies this manuscript include Supplementary Table S1–S3, Supplementary Figs. S1–S16 and Supplementary Methods.

Supplementary Table S1. Average cell count of penetrating cells in the transwell cell migration and invasion assays.

	Average cell count of invading/migrating cells					
Fig. 1b Invasion	CTLi (H292)	288.6 ± 9.3	CTLi (H292)	288.6 ± 9.3	CTLi (H292)	318.0 ± 15.8
	TRPM7i	102.0 ± 12.9[a]	ORAI1i	186.6 ± 15.1[a]	STIM1i	250.6 ± 15.7[a]
Fig. 1d Invasion	CTLi (H460)	405.7 ± 56.1	CTLi (A549)	481.2 ± 7.8	CTLi (H23)	194.8 ± 1.6
	TRPM7i	204.3 ± 18.2[a]	TRPM7i	218.2 ± 26.8[a]	TRPM7i	56.4 ± 11.1[a]
Fig. 2b	Invasion (H292)	0 µM	385.7 ± 22.1	Migration (H292)	0 µM	410.0 ± 37.3
		10 µM	166.9 ± 47.6[b]		10 µM	284.5 ± 19.6[b]
		20 µM	83.5 ± 12.4[b]		20 µM	159.5 ± 7.4[b]
		25 µM	63.0 ± 24.5[b]		25 µM	108.3 ± 16.5[b]

Penetrating cells were stained by Hoechst 33342 at 48 h and visualized and scored under a fluorescence microscope. Data are means ± s.d. from three or more independent experiments. [a]$p < 0.05$ versus CRISPR/Cas9 control CTLi cells; two-sided Student's *t*-test. [b]$p < 0.05$ versus nontreated cells; two-sided Student's *t*-test.

Supplementary Table S2. Percentage of change in the wound space (wound closure) in the wound healing assay.

			Percentage of change in the wound space			
Fig. 2e and f	NCI-H292	0 µM	84.2 ± 2.9	NCI-H460	0 µM	94.5 ± 5.4
		10 µM	57.7 ± 4.8[a]		10 µM	51.2 ± 2.3[a]
		20 µM	32.9 ± 3.8[a]		20 µM	33.4 ± 4.7[a]
		25 µM	19.9 ± 1.0[a]		25 µM	19.7 ± 1.0[a]
Fig. 2g	CTL (Prn4)	84.7 ± 6.2	CTL (ECL12)	96.8 ± 2.5	CTL (ECL17)	89.5 ± 2.0
	2-APB at 20 µM	48.6 ± 3.8[a]	2-APB at 20 µM	66.1 ± 4.1[a]	2-APB at 20 µM	53.0 ± 4.2[a]
	2-APB at 25 µM	34.7 ± 4.7[a]	2-APB at 25 µM	55.9 ± 4.6[a]	2-APB at 25 µM	44.5 ± 1.5[a]
Fig. 3c and d	NCI-H292	CTLi	92.7 ± 0.8	NCI-H460	CTLi	97.5 ± 2.7
		TRPM7i	29.0 ± 0.6[b]		TRPM7i	44.5 ± 1.5[b]
Fig. 3e	NCI-H292	CTLi	92.0 ± 0.83			
		OGTi	42.0 ± 4.0[b]			
Fig. 4a	CTL	81.8 ± 2.6		Fig. 4b	CTLi	88.1 ± 5.3
	TMG	91.3 ± 10.3			CTLi + TMG	102.4 ± 7.7
	2-APB	26.6 ± 2.4[a]			TRPM7i	38.1 ± 2.2[b]
	2-APB + TMG	53.9 ± 5.7[a,c]			TRPM7i + TMG	65.1 ± 4.31[a,c]
Fig. 5g	CTLi	84.6 ± 5.6		Fig. 5h	CTLi	84.6 ± 5.6
	CAV1i	38.2 ± 8.2[b]			MYCi	32.6 ± 9.5[b]
	CAV1i + TMG	67.5 ± 7.4[b,c]			MYCi + TMG	61.5 ± 4.7[b,c]

Wound spaces were made and measured at 0 h and 24 h (Prn4, ECL12, ECL17) or 48 h and percentage of change in the space was calculated. Data are means ± s.d. from three or more independent experiments. [a]$p < 0.05$ versus nontreated cells; two-sided Student's *t*-test or one-way ANOVA with Bonferroni post-test (Fig. 4a and 4b). [b]$p < 0.05$ versus CRISPR/Cas9 control CTLi cells; two-sided Student's *t*-test. [c]$p < 0.05$ versus 2-APB-treated cells or TRPM7i cells alone; two-sided Student's *t*-test or one-way ANOVA with Bonferroni post-test (Fig. 5g and 5h).

Supplementary Table S3. Clinical characteristics of patient-derived primary cancer cell cultures and treatment summation.

Designation	Age (yr)	Sex	Histological subtype	Molecular characteristics	Treatment
Prn4	69	Female	Adenocarcinoma	*EGFR* mutation L858R, T790M	1L Carboplatin/gemcitabine<br>2L Docetaxel<br>3L Erlotinib<br>4L Pemetrexed
ECL12	71	Female	Squamous cell carcinoma		Naïve for treatment
ECL17	74	Male	Adenocarcinoma		1L Ccarboplatin/paclitaxel/ bevacizumab

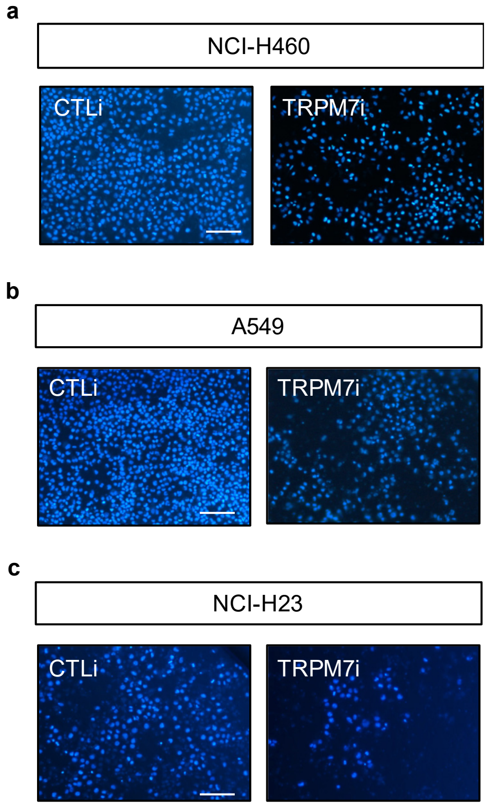

Supplementary Fig. S1. Inhibition of TRPM7 suppresses cell invasion in multiple NSCLC cell lines. NCI-H460, A549 and NCI-H23 cells were genetically knocked down with TRPM7 (TRPM7i) or control (CTLi) gRNAs in CRISPR/Cas9 system and cell invasion was evaluated by transwell assay. Representative fluorescence micrographs of invading cells stained with

Hoechst33342 dye comparing CTLi and TRPM7i cells in NCI-H460 (**a**), A549 (**b**) and NCI-H23 (**c**) cells are shown. Scale bar = 100 µm.

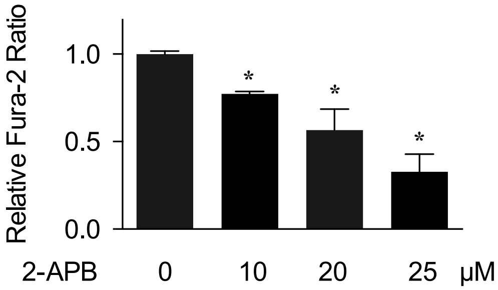

Supplementary Fig. S2. Quantitative analysis of intracellular Ca^{2+} in response to 2-APB treatment (0–25 µM) in NCI-H292 cells by a fluorescence plate reader using Fura-2 AM as a specific probe. Fura-2 signals were measured at 340/510 and 380/510 nm and reported as relative Fura-2 ratio to nontreated control. Data are mean ± s.d. (n = 3). $^{*}p < 0.05$ versus nontreated cells; two-sided Student's t test.

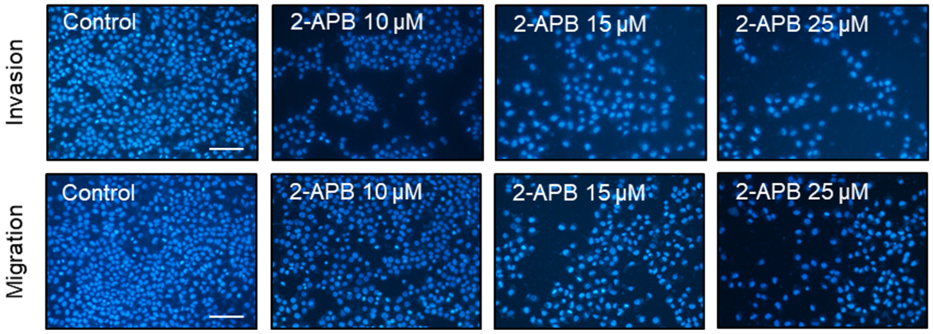

Supplementary Fig. S3. Effects of TRPM7 inhibition by 2-APB on cell motility.

NSCLC NCI-H292 cells were treated with 2-APB (0–25 µM) and cell invasion and migration were evaluated by transwell asssay at 48 h post incubation. Representative micrographs comparing invading and migrating cells stained with Hoechst33342 dye are shown. Scale bar = 100 µm.

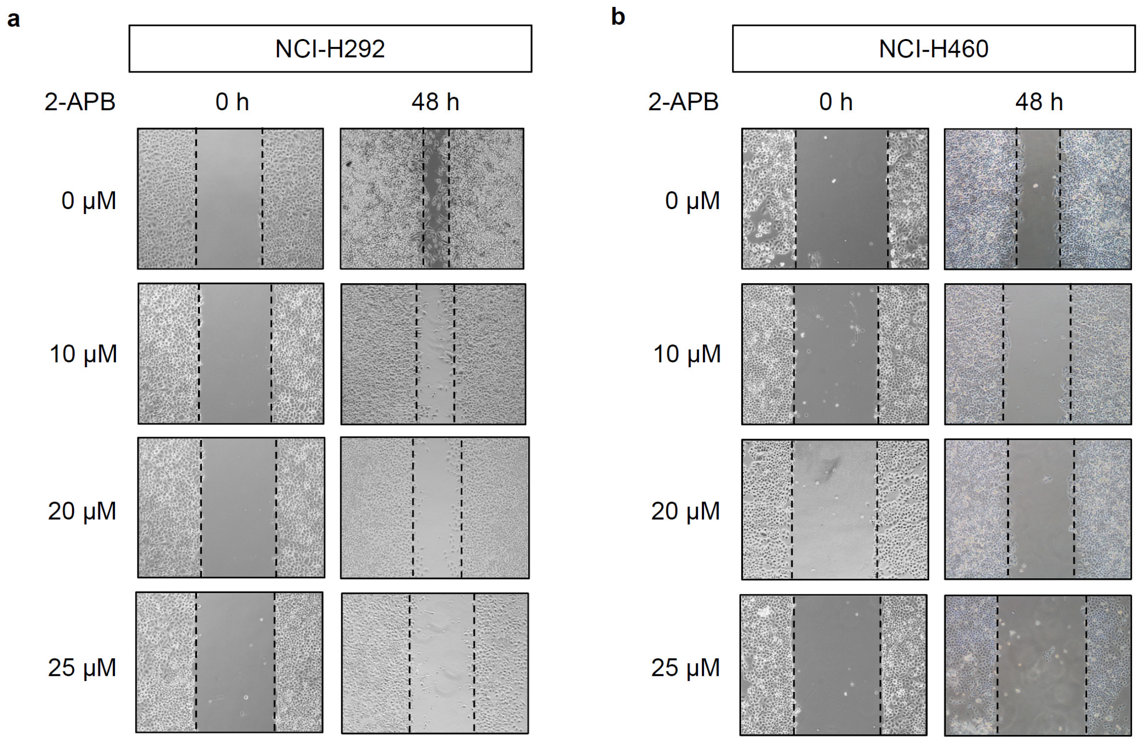

Supplementary Fig. S4. Effects of TRPM7 inhibition by 2-APB on cell migration by wound healing assay. NCI-H292 (**a**) and NCI-H460 (**b**) cells were treated with 2-APB (0–25 μM) and wound space as shown in representative micrographs was visualized at 0 and 48 h.

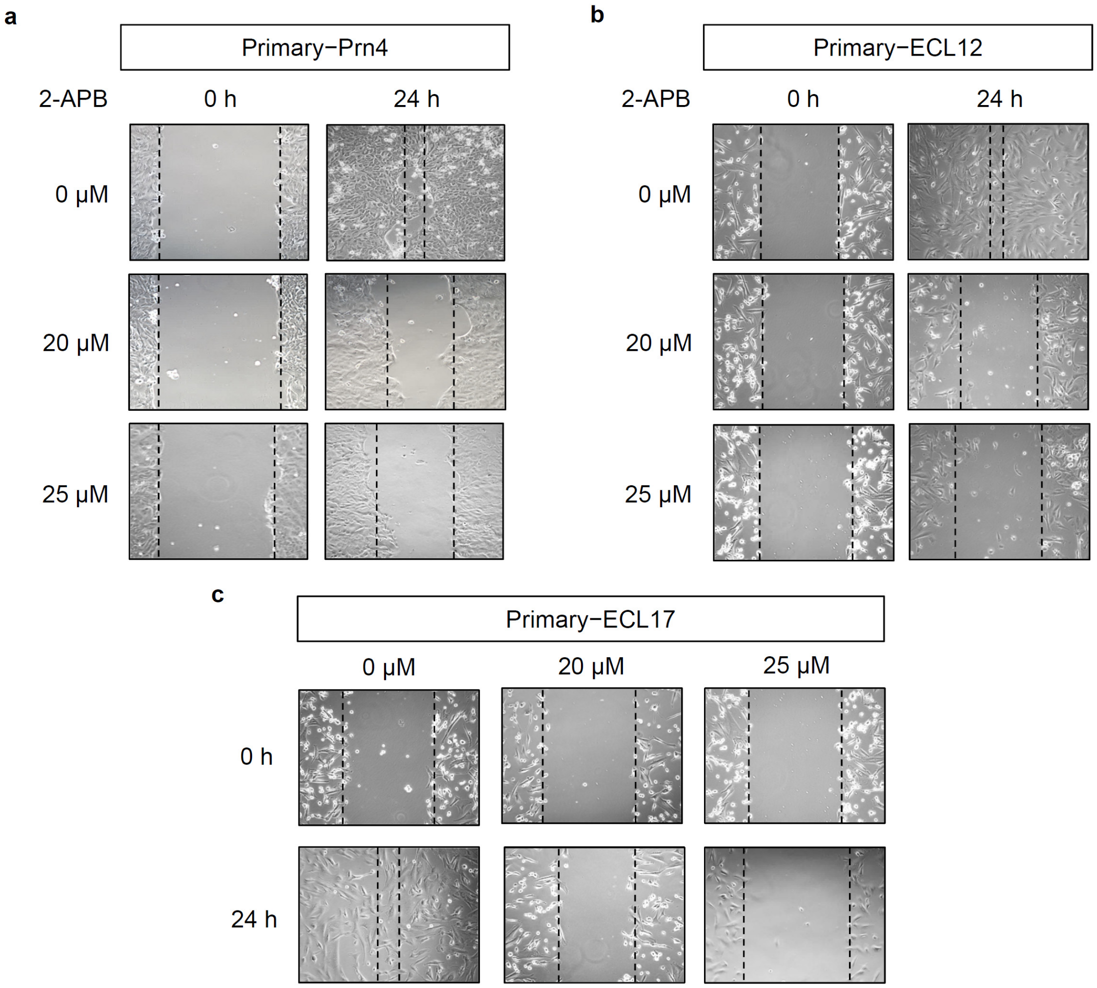

Supplementary Fig. S5. Effects of TRPM7 inhibition by 2-APB on cell migration of primary lung cancer cells as evaluated by wound healing assay. Primary Prn4 (**a**), ECL12 (**b**) and ECL17 (**c**) cells were treated with 2-APB (0–25 μM) and wound space as shown in representative micrographs was visualized at 0 and 24 h.

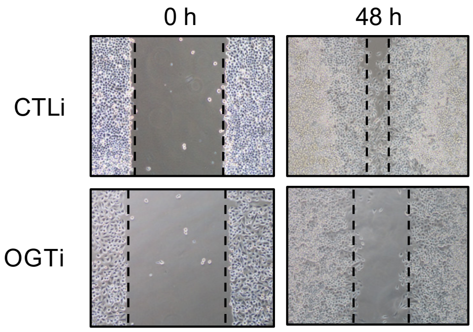

Supplementary Fig. S6. Inhibition of *O*-GlcNAcylation decreases NSCLC cell migration as evaluated by wound healing assay. NCI-H292 cells were genetically knocked down with OGT (OGTi) or control (CTLi) gRNAs in CRISPR/Cas9 system and cell migration was evaluated by wound healing assay. Representative micrographs of wound space are shown at 0 and 48 h. (**b**)

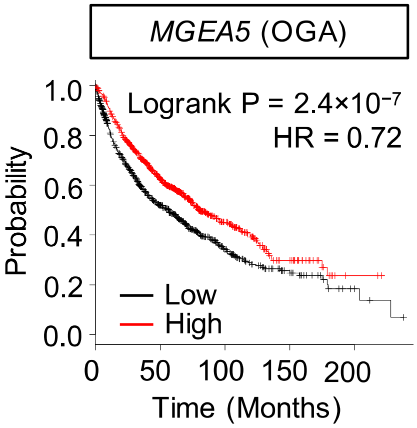

Supplementary Fig. S7. Kaplan–Meier survival curve of patients with NSCLC, segregated according to high (red) or low (black) expression of *MGEA5* (encoding OGA), obtained from Kaplan-Meier Plotter (http://kmplot.com/analysis/).

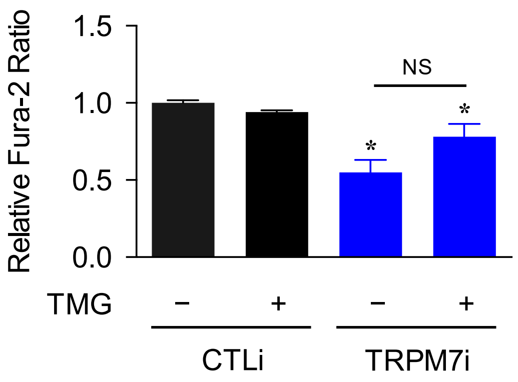

Supplementary Fig. S8. Quantitative analysis of intracellular Ca^{2+} in TRPM7 knockdown (TRPM7i) and control (CTLi) NCI-H292 cells in the presence or absence of thiamet G (TMG; 10 μM) by a fluorescence plate reader using Fura-2 AM as a specific probe. Flura-2 signals were measured at 340/510 and 380/510 nm and reported as relative Fura-2 ratio to nontreated CTLi cells. Data are mean ± s.d. (n = 3). $^{*}p < 0.05$ versus nontreated CTLi cells; one-way ANOVA with Bonferroni post-test. NS, not significance versus TRPM7i cells; one-way ANOVA with Bonferroni post-test.

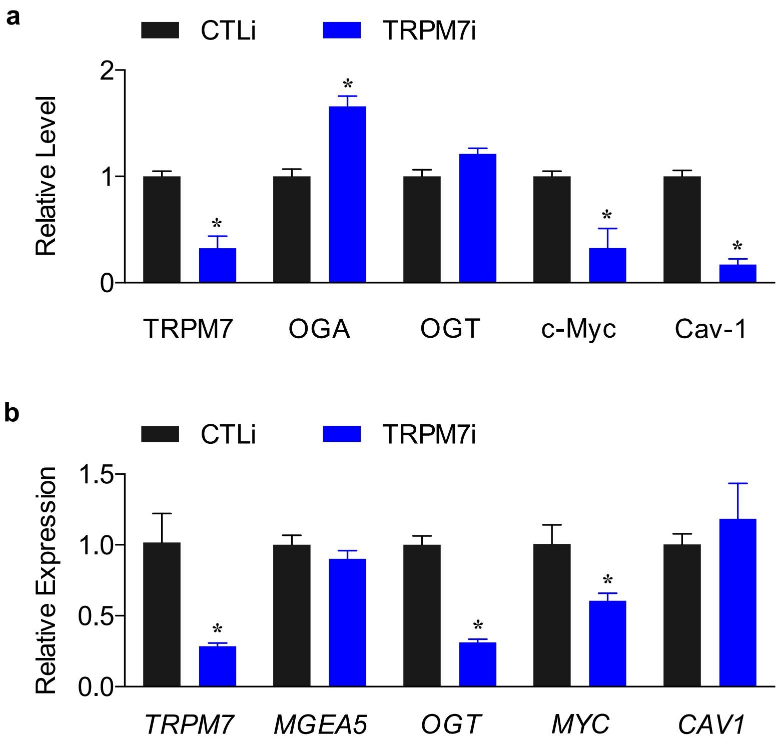

Supplementary Fig. S9. (a) Quantitative analysis of TRPM7, OGA, OGT, c-Myc and Cav-1 levels by densitometry in correspond to Western blot analysis in Fig. 4c. Data are mean ± s.d. (n = 3). $^{*}p < 0.05$ versus CTLi cells; two-sided Student's *t* test. (**b**) Quantitative real-time PCR of *TRPM7*, *MGEA5* (encoding OGA), *OGT*, *MYC* and *CAV1* mRNA expression. Data are mean ± s.d. (n = 3). $^{*}p < 0.05$ versus CTLi cells; two-sided Student's *t* test.

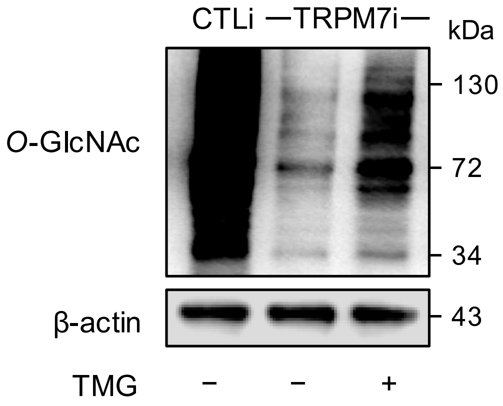

Supplementary Fig. S10. Western blot analysis of global *O*-GlcNAcylation in response to thiamet G in NCI-H292 cells upon TRPM7 inhibition. NCI-H292 cells were treated with thiamet G (TMG; 10 μM) that causes hyper-*O*-GlcNAcylation for 24 h and *O*-GlcNAc level was determined by Western blotting.

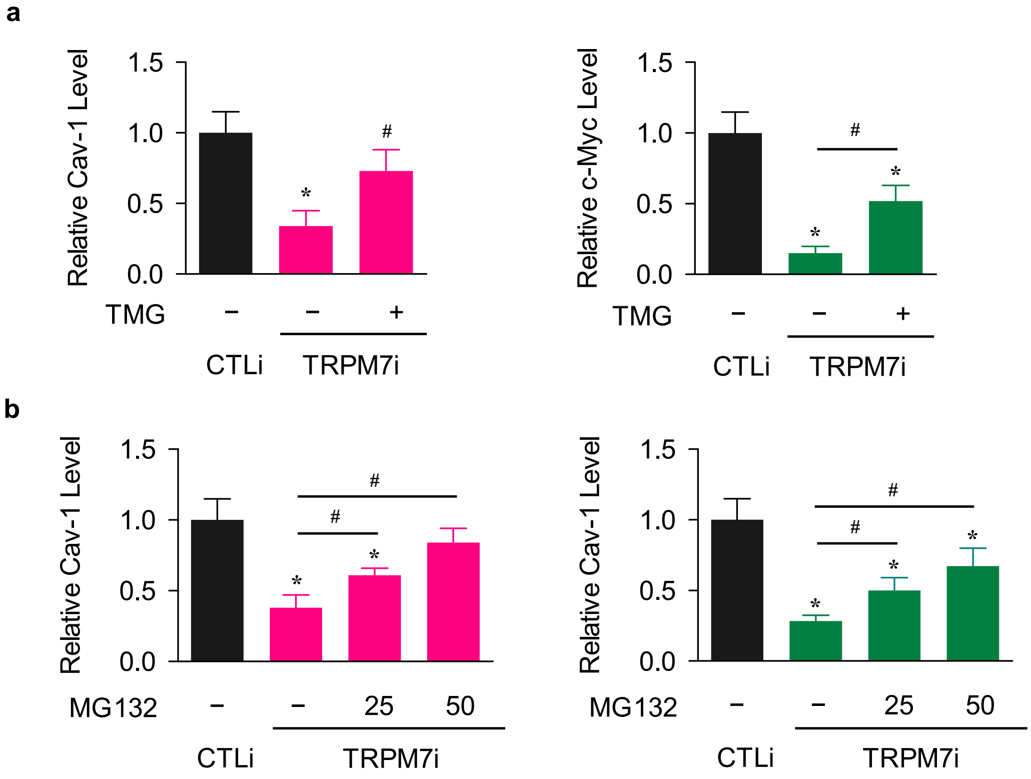

Supplementary Fig. S11. TRPM7 mediates Cav-1 and c-Myc via *O*-GlcNAcylation and ubiquitin-mediated proteasomal degradation. Quantitative analysis of Cav-1 and c-Myc levels by

densitometry in TRPM7i cells with or without OGA inhibitor thiamet G (TMG; 10 µM) (**a**) or proteasomal inhibitor MG132 (25−50 µM) (**b**) in correspond to the Western blot analysis in Fig. 4e and f. Data are mean ± s.d. (n = 3). $^{*}p < 0.05$ versus CTLi cells; one-way ANOVA with Bonferroni post-test. $^{\#}p < 0.05$ versus TRPM7i cells without TMG or MG132; one-way ANOVA with Bonferroni post-test.

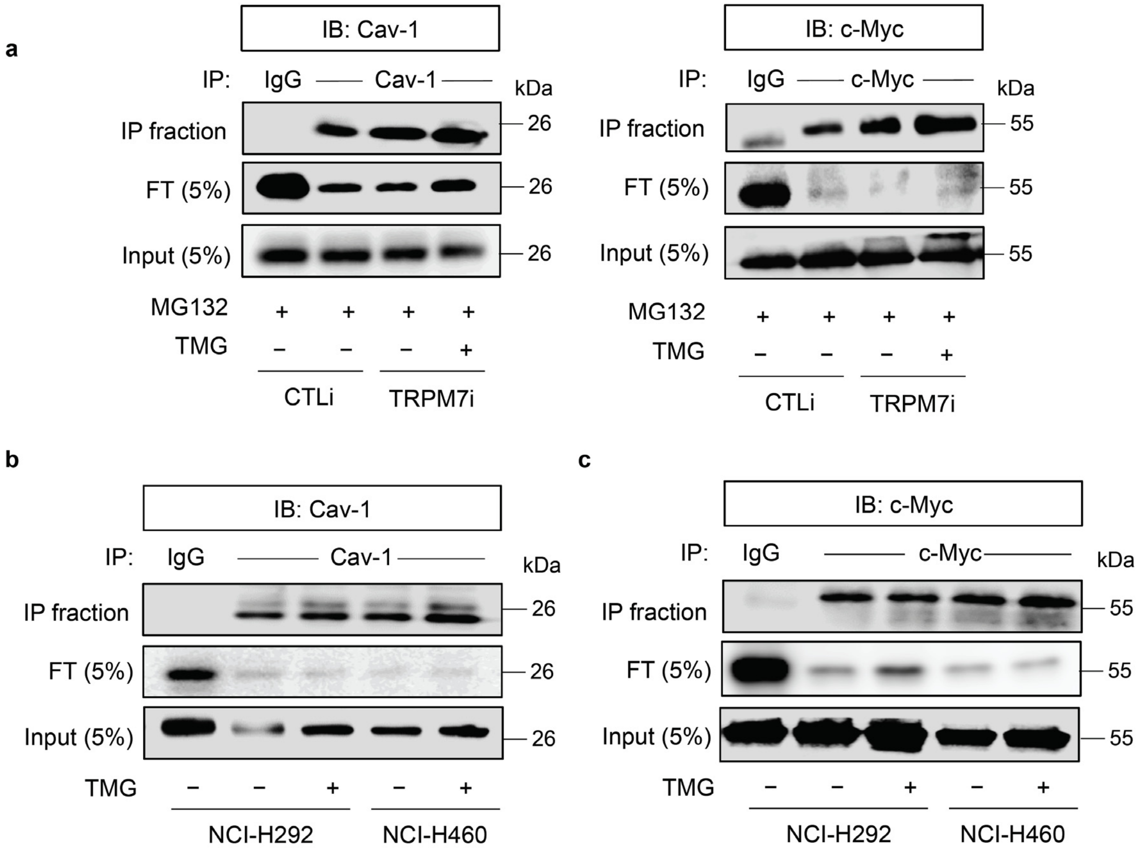

Supplementary Fig. S12. Western blot analysis of Cav-1 and c-Myc in different fractions from immunprecipitation experiments, including immunoprecipitated (IP), flow through (FT) and input, in correspond to the data in Fig. 5b–d.

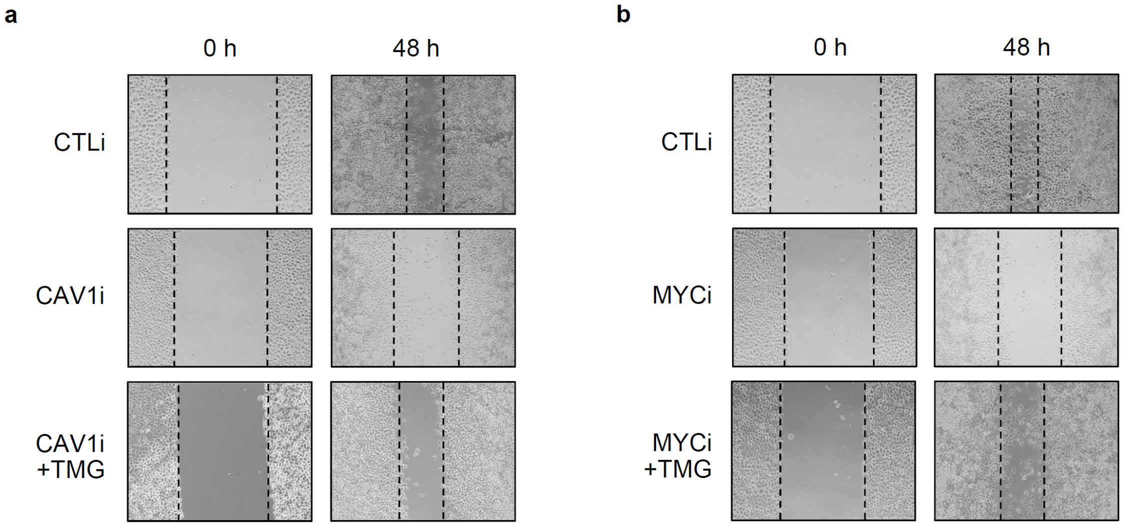

Supplementary Fig. S13. Effects of thiamet G on cell migration upon Cav-1 and c-Myc inhibition as evaluated by wound healing assay. Cav-1 and c-Myc were inhibited by using shRNAs against *CAV1* (a) and *MYC* (b) in NCI-H292 cells, treated with thiamet G (TMG; 10 μM) and wound space as shown in representative micrographs was visualized at 0 and 48 h in comparison to CTLi cells.

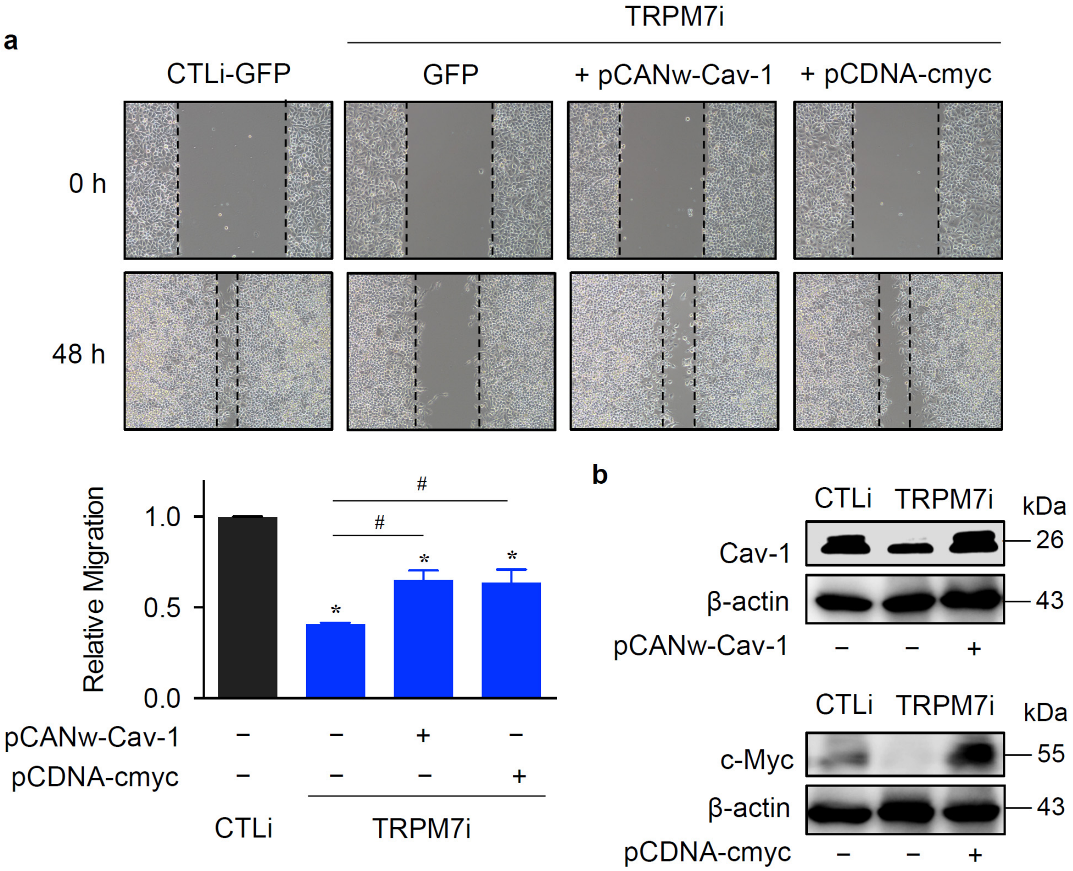

Supplementary Fig. S14. Effects of Cav-1 and c-Myc overexpression on cell migration upon TRPM7 inhibition as evaluated by wound healing assay. (a) NCI-H292 TRPM7i cells were transfected with Cav-1 (pCANw-Cav-1) or c-Myc (pCDNA-cmyc) overexpression plasmid and analyzed for cell migration by wound healing assay in comparison to CTLi cells. Representative micrographs of wound space visualized at 0 and 48 h are shown. Data are mean ± s.d. (n = 3). $^{*}p < 0.05$ versus CTLi cells; one-way ANOVA with Bonferroni post-test. $^{\#}p < 0.05$ versus TRPM7i cells without pCANw-Cav-1 or pCDNA-cmyc plasmid; one-way ANOVA with Bonferroni post-test. (b) Western blot analysis of Cav-1 and c-Myc levels in Cav-1 and c-Myc-overexpressed TRPM7i cells.

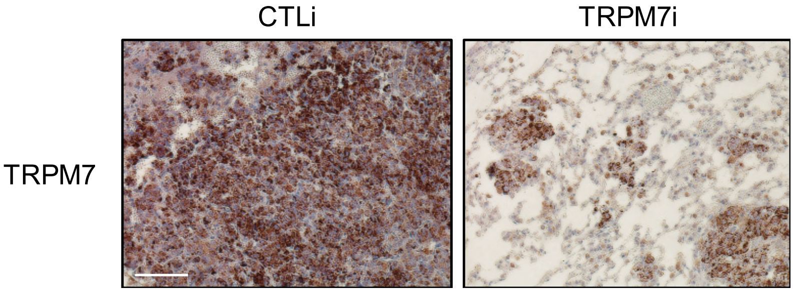

Supplementary Fig. S15. Immunohistochemistry analysis of TRPM7 in isolated lungs from mice bearing CTLi and TRPM7i NCI-H292 cells. Scale bar = 100 μm.

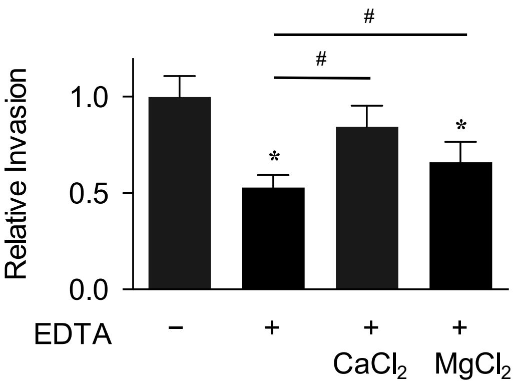

Supplementary Fig. S16. Effects of ion chelation and extracellular Ca^{2+} and Mg^{2+} on cell invasion. NCI-H292 cells were treated with EDTA (1.5 mM), followed by an addition of equal concentration of $CaCl_2$ (1.5 mM) or $MgCl_2$ (1.5 mM) and cell invasion was evaluated as by transwell assay at 48 h. Data are mean ± s.d. (n = 3). $^{*}p < 0.05$ versus nontreated cells; one-way ANOVA with Bonferroni post-test. $^{\#}p < 0.05$ versus EDTA-treated cells; one-way ANOVA with Bonferroni post-test.

SUPPLEMENTARY METHODS

In vivo bioluminescence imaging of tumors in mice

In vivo bioluminescence imaging does not require removal of tissues to track tumor growth, thus it allows repeated measurements to be made in the same mice at multiple time points. By imaging the signals at the time of inoculation (baseline), each mice serves as its own control. The noninvasive nature of *in vivo* imaging also provides a valuable approach to minimize potential pain and distress, and hence it could both reduce and refine the use of animals. Male NOD/SCID gamma mice, strain NOD.Cg-$Prkdc^{scid}$ $Il2rg^{tm1Wjl}$/SzJ (NSG; WVU Transgenic Animal Core Facility), aged 6–8 weeks, median weight 26 g were maintained under pathogen-free conditions within the institutional animal facility and assigned randomly into two experimental groups. Food and tap water were given ad libitum. Cells were first labeled with UBC-RFP-T2A-Luciferase dual reporter and RFP-positive cells were sorted to enrich the luciferase signal. After tail vein injection of luciferase-labeled tumor cells, mice were imaged over time at the time of inoculation (W0), and 1 (W1), 2 (W2) and 3 (W3) weeks post injection using IVIS imaging (PerkinElmer, Waltham, MA). D-luciferin, Firefly, potassium salt (Caliper Life Sciences, Hopkinton, MA) was prepared as a stock solution at 15 mg/mL in DPBS. Mice were received an intraperitoneal injection of luciferin (150 μg per kg of mouse body weight or 100 μL of luciferin stock per 10 g of body weight) 5−10 min before imaging and were anesthetized with 4% isoflurane gas at the induction in Plexiglas anesthesia box. After mice were fully anesthetized, they were transferred from the box to the nose cones attached to the imaging chamber with 2% isoflurane gas during imaging procedure. The mice were imaged for 5 min and returned to their cage where they awoke quickly. At the end of experiments, mice were euthanized with cervical dislocation under carbon dioxide inhalation and the lungs, liver, brain, heart, kidneys and spleens, were dissected and further analyzed for tumor histopathology.

Tumor histopathology

Isolated lung tissues from tumor-bearing mice were formalin-fixed and paraffin-embedded. The specimens were cut into 5-µm sections and stained with hematoxylin and eosin to define the tumor morphology and cellular structure within the lungs. Tissue sectioning and hematoxylin and eosin staining were performed at the Pathology Laboratory for Translational Medicine, WVU Cancer Institute. The presence of multinucleated cells and condensation of heterochromatin (hematoxylin staining) were considered as cancer-specific patterns. Immunohistochemical (IHC) staining was performed at the Immunopathology Laboratory, Faculty of Medicine Siriraj Hospital using Dako Envision FLEX reagents (Dakocytomation Company, Denmark). The primary antibodies used for IHC analysis included anti-TRPM7 (abcam #ab262698), anti-Cav-1 (Santacruz Biotechnology, #sc-894) and anti-c-Myc (Cell Signaling Technology, #13987) at 1:200 dilution, while the secondary antibody was SM802 Envision FLEX/HRP. Colorimetric detection was performed by using SM803 FLEX DAB+ Sub-Chromo and counterstained with hematoxylin.
